# Contribution of Noncentrosomal Microtubules to Spindle Assembly in *Drosophila* Spermatocytes

**DOI:** 10.1371/journal.pbio.0020008

**Published:** 2004-01-20

**Authors:** Elena Rebollo, Salud Llamazares, José Reina, Cayetano Gonzalez

**Affiliations:** **1**Cell Biology and Biophysics Programme, European Molecular Biology LaboratoryHeidelbergGermany

## Abstract

Previous data suggested that anastral spindles, morphologically similar to those found in oocytes, can assemble in a centrosome-independent manner in cells that contain centrosomes. It is assumed that the microtubules that build these acentrosomal spindles originate over the chromatin. However, the actual processes of centrosome-independent microtubule nucleation, polymerisation, and sorting have not been documented in centrosome-containing cells. We have identified two experimental conditions in which centrosomes are kept close to the plasma membrane, away from the nuclear region, throughout meiosis I in *Drosophila* spermatocytes. Time-lapse confocal microscopy of these cells labelled with fluorescent chimeras reveals centrosome-independent microtubule nucleation, growth, and sorting into a bipolar spindle array over the nuclear region, away from the asters. The onset of noncentrosomal microtubule nucleation is significantly delayed with respect to nuclear envelope breakdown and coincides with the end of chromosome condensation. It takes place in foci that are close to the membranes that ensheath the nuclear region, not over the condensed chromosomes. Metaphase plates are formed in these spindles, and, in a fraction of them, some degree of polewards chromosome segregation takes place. In these cells that contain both membrane-bound asters and an anastral spindle, the orientation of the cytokinesis furrow correlates with the position of the asters and is independent of the orientation of the spindle. We conclude that the fenestrated nuclear envelope may significantly contribute to the normal process of spindle assembly in *Drosophila* spermatocytes. We also conclude that the anastral spindles that we have observed are not likely to provide a robust back-up able to ensure successful cell division. We propose that these anastral microtubule arrays could be a constitutive component of wild-type spindles, normally masked by the abundance of centrosome-derived microtubules and revealed when asters are kept away. These observations are consistent with a model in which centrosomal and noncentrosomal microtubules contribute to the assembly and are required for the robustness of the cell division spindle in cells that contain centrosomes.

## Abstract 2

Time-lapse confocal microscopy reveals a potential role for noncentrosomal microtubules nucleated near the nuclear envelope in spindle assembly in Drosophila spermatocytes

## Introduction

Two different pathways of spindle assembly are known to operate in the animal kingdom. The first, observed in somatic as well as in male germline cells, requires the microtubule organising activity of centrosomes (Compton 2000; Bornens 2002). The second, restricted to female germline and some embryonic cells that lack centrosomes, is thought to depend upon the microtubule stabilisation and organisation activity of the chromosomes themselves (McKim and Hawley 1995; de Saint Phalle and Sullivan 1998; reviewed in Karsenti and Vernos 2001). Centrosome-independent microtubule growth and sorting into a bipolar spindle have been observed in vitro around chromatin-coated beads in *Xenopus* egg extracts (Heald et al. 1996). Moreover, some experimental data suggest that a centrosome-independent pathway for spindle assembly also exists in somatic cells (Bonaccorsi et al. 1998, 2000; Megraw et al. 1999, 2001; Vaizel-Ohayon and Schejter 1999; Khodjakov et al. 2000; Hinchcliffe et al. 2001; reviewed in Raff 2001). It is generally assumed that the microtubules that build these acentrosomal spindles originate over the chromatin. However, so far, the actual process of centrosome-indepen dent microtubule nucleation, polymerisation, and sorting into a bipolar spindle has not been documented in any of the centrosome-containing cell lineages of a living animal.

The problem in visualising such microtubules of noncentrosomal origin when centrosomes are present is a technical one. In *Drosophila*, as in most animal cells, at the onset of cell division the two segregated pairs of centrosomes have a strong microtubule organising activity (Tates 1971; Church and Lin 1982; Cenci et al. 1994). Consequently, as soon as the nuclear envelope (NE) breaks down, numerous microtubules invade the nuclear region, making it extremely difficult to single out any noncentrosomal microtubules that might be present. To circumvent this limitation, we have taken advantage of two experimental conditions that inhibit the natural process of centriole migration from the plasma membrane to the interior of the cell that takes place at the onset of meiosis in *Drosophila* spermatocytes. Under such conditions, the centrosomes organise asters, but these are kept at the plasma membrane, away from the nuclear region. In these cells, microtubules can be seen to grow from the remnants of the fenestrated NE and to assemble into anastral bipolar spindles in a centrosome-independent manner. We propose that these spindle-shaped arrays correspond to a subset of microtubules that are normally present in the spindles of wild-type cells.

## Results

### Centriole Migration towards the Nucleus in *Drosophila* Spermatocytes Requires Microtubules and the Function of *asp*

**Figure 1:**
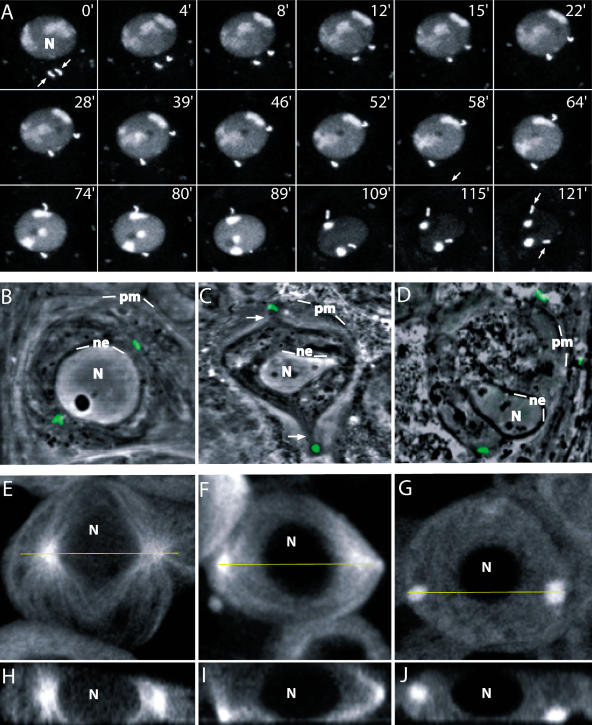
Centriole Migration in Primary Spermatocytes (A) Time-lapse series of confocal images from a wild-type primary spermatocyte expressing GFP-PACT (centrioles) and His2AvD–GFP (chromosomes). The centrioles (arrows) can be seen moving away from the plasma membrane (0) towards the nucleus (N) and then migrating diametrically apart as the chromatin condenses. The chromosomes are fully condensed at timepoint 121 min. (B–D) The two centriole pairs (green) projected over the phase-contrast view (grey) can be seen close to the fenestrated NE and away from the plasma membrane (pm) in control cells (B), while they remain plasma membrane-bound in *asp* (C) and in colcemid-treated wild-type cells (D). In *asp* spermatocytes (C), the position of the membrane-bound centrioles correlates tightly with the pointed end of phase-dark protrusions (arrows) that are not present in colcemid-treated cells. These reflect the distribution of phase-contrast membranes known to overlap microtubules in these cells. (E–J) XY projections (E–G) and their corresponding optical sections (H–J) of control (E and H), *asp* (F and I), colcemid-treated spermatocytes (G and J) expressing an endogenous GFP–α-tubulin confirm that the two major MTOCs in control cells are close to the nucleus, but remain near the plasma membrane in the two experimental conditions. MTOC activity in colcemid-treated spermatocytes was assayed following a 1-s pulse of 350 nm light to inactivate the drug, thus allowing microtubule regrowth. The yellow bar in the XY projections (E–G) marks the position of the corresponding XZ optical sections (H–J).

Studies based on electron microscopy (Tates 1971) had shown that soon after the last round of mitotic divisions that precede meiosis in *Drosophila* spermatocytes, the centrioles migrate towards the periphery of the cell and position themselves underneath the plasma membrane. The same studies revealed that shortly before the onset of prometaphase I, the centrioles are found again close to the nuclear membrane, thus strongly suggesting that they migrate back near the nucleus in preparation for meiosis. Using an endogenously expressed centriolar green fluorescent protein (GFP) marker, we have been able to demonstrate such migration in living spermatocytes (Figure 1A; Video 1). The entire process takes about 2 h. Initially, the two centriolar pairs move towards the nucleus and start to migrate apart as they approach the nuclear membrane. They finally position themselves at opposite sides of the nucleus, about 30 min before the onset of NE breakdown (NEB).

We have identified two experimental conditions that inhibit centriole movement, back from the plasma membrane in *Drosophila* spermatocytes. The first one is mutation in the gene abnormal spindle, *asp* (Ripoll et al. 1985; Casal et al. 1990; Gonzalez et al. 1990; Saunders et al. 1997; do Carmo Avides and Glover 1999; Wakefield et al. 2001; Riparbelli et al. 2002). In contrast to wild-type control cells (Figure 1B), the two pairs of centrioles in *asp^E3^/asp^L1^* spermatocytes at late prophase are still located at the plasma membrane (Figure 1C), where they remain throughout meiosis. Centriole migration back towards the NE can also be inhibited by microtubule depolymerisation. Like in *asp* mutant spermatocytes, the centrioles of wild-type spermatocytes exposed to the microtubule-depolymerising drug colcemid remain close to the plasma membrane throughout meiosis (Figure 1D).

Top (Figure 1F) and lateral (Figure 1I) views of living *asp* mutant spermatocytes expressing a GFP–α-tubulin fusion reveal that the microtubule organising centres (MTOCs) are found at the periphery of these cells. The MTOCs of control cells at this stage can be seen near the nuclear membrane (Figure 1E and 1H). These observations strongly suggest that the membrane-bound centrioles observed in *asp* mutant spermatocytes are associated to active centrosomes that retain MTOC activity. This conclusion is further substantiated by the localisation of the pericentriolar material (PCM) marker γ-Tub23C (Zheng et al. 1991; Sunkel et al. 1995) around the membrane-bound centrioles, coinciding with the position of the MTOCs (data not shown). The same applies to cells in which centriole migration is inhibited by colcemid. Immediately after a short pulse of 350 nm UV light to inactivate the drug (W. E. Theurkauf, personal communication), two asters are organised around the membrane-bound centrioles in these cells (Figure 1G and 1J).

### Anastral Spindles Are Assembled When the Centrosomes Are Kept Membrane Bound

**Figure 2:**
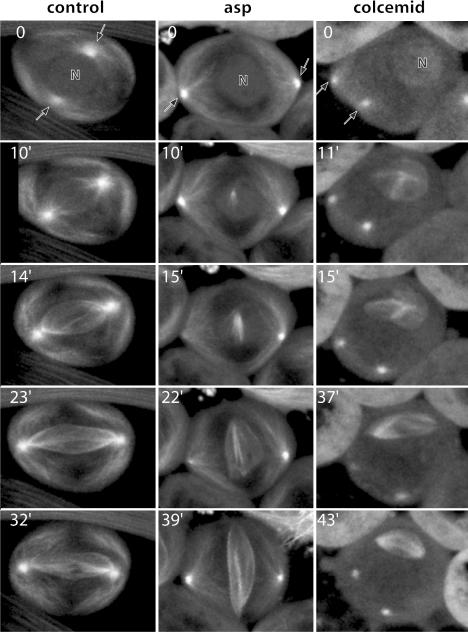
Time-Lapse Series of Meiosis Progression in Control, *asp*, and Colcemid-Treated Spermatocytes Timepoint 0 coincides with the time of NEB revealed by the sudden entry of GFP signal into the nucleus. In control cells (Video 2), microtubules are mainly organised around the centrosomes (arrows). However, when centrosomes are kept away from the nuclear region by mutation in *asp* (Video 3) or colcemid treatment (Video 4), microtubule nucleation and growth are clearly revealed over the nuclear region (N), well away from the centrosomes. Such noncentrosomal microtubules may form bundles that eventually are sorted into spindlelike bipolar microtubule arrays. Microtubules were labelled with an endogenous GFP–α-tubulin fusion.

Inhibition of centrosome migration back from the plasma membrane in *Drosophila* spermatocytes offers an unprecedented opportunity to assay centrosome-independent microtubule polymerisation during spindle assembly in the cells of a living animal that contain centrosomes. Therefore, we decided to follow microtubules by time-lapse confocal microscopy in *asp* and colcemid-treated cells that expressed a GFP–α-tubulin fusion, as they went through meiosis. At the onset of prometaphase, the NE becomes fenestrated, but does not disappear in *Drosophila* (Tates 1971; Stafstrom and Staehelin 1984; Church and Lin 1985). This partial NEB can be readily identified by the sudden entry of GFP–α-tubulin into the nuclear region (Figure 2, timepoint 0; Video 2). In control cells, microtubule polymerisation and organisation are largely concentrated around the centrosomes (Church and Lin 1982; Cenci et al. 1994). Consequently, the abundance of these microtubules makes it extremely difficult to determine the possible contribution of any centrosome-independent microtubule polymerisation activity to spindle assembly (Figure 2, control, 10 min to 32 min; Video 2). Microtubule organisation is significantly different in the case of *asp* mutant spermatocytes. At the time of NEB, the membrane-bound centrosomes can be seen organising the two asters at a significant distance from the nucleus, which is kept clear from astral microtubules (Figure 2; Video 3). Around 10 min after NEB, a distinct focus of microtubule polymerisation appears within the nuclear region, away from the asters (Figure 2, *asp*, 10 min; Video 3). It gives rise to a few bundles (Figures 2, 15 min) that grow (Figure 2, 22 min) and get organised into a bipolar spindle-shaped microtubule array that in 28% (*n* = 43) of the cells is anastral and establishes no contact with the membrane-bound centrosomes (Figure 2, 39 min). The remaining 72% was accounted for by cells in which, despite the distance, microtubules from one or both asters reach the spindle so that spindle poles and asters were aligned. Although the acentrosomal origin of the spindle microtubules in these cells is fairly convincing, only those cells that assembled truly anastral spindles that remained so throughout meiosis were considered as cases of noncentrosomal spindle assembly.

These observations strongly suggested that microtubules can nucleate in a centrosome-independent manner and assemble a spindle-like array in *Drosophila* spermatocytes. The question remained open, however, as to whether such anastral structures could not simply be a consequence of mutation in *asp* itself. To rule out such a possibility, we followed spindle assembly in wild-type spermatocytes in which centrosomes had been kept membrane-bound by colcemid treatment. The results were strikingly similar to those observed in *asp* cells. Seconds after colcemid inactivation, the cortex-bound position of the centrosomes is revealed by the growing asters that were not visible before (Figure 2, colcemid inactivation, 0 timepoint; Video 4). Like in *asp* mutant spermatocytes, microtubules can clearly be seen to nucleate over the nuclear region, well away from the asters (Figure 2, 11 min), grow (Figure 2, 15 min), and get sorted (Figure 2, 37 min and 43 min) into anastral bipolar arrays. Of the colcemid-treated cells studied (*n* = 32), 22% behaved like the cell shown in Figure 2. The remaining cells assembled more than one spindle, multipolar spindles, or spindles that were connected to one of the asters.

### The Nucleation of Noncentrosomal Microtubules in Spermatocytes with Membrane-Bound Centrosomes Has a Late Onset

**Figure 3:**
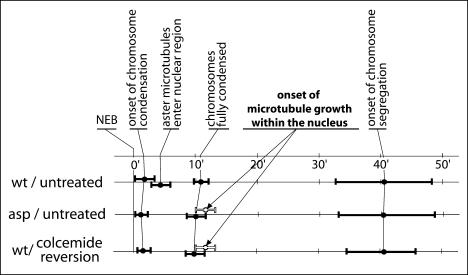
The Timing of Noncentrosomal Microtubule Nucleation Referred to NEB (timepoint 0), the timing of chromosome condensation and of onset of chromosome segregation is essentially identical in control, *asp*, and colcemid-treated spermatocytes. In control spermatocytes, aster microtubules can be seen entering the nuclear region 3–6 min after NEB. They do not in *asp* or following colcemid treatment. In these two cases, however, centrosome-independent microtubule polymerisation can be seen over the nuclear region. It starts between 9 and 13 min after NEB, coinciding with or very shortly after the end of chromosome condensation.

We then decided to time the onset of anastral microtubule nucleation. The timing of the main landmarks of meiosis progression in control, *asp*, and colcemid-treated spermatocytes is summarised in Figure 3. In control spermatocytes, chromosome condensation starts within 2 min after NEB, and the first centrosomal microtubules enter the nuclear region shortly afterwards. Chromosome condensation is completed between 10 and 12 min after NEB. In agreement with previous reports, anaphase onset takes place between 32 and 47 min after NEB (Church and Lin 1985; Rebollo and Gonzalez 2000; Savoian et al. 2000). Remarkably, the timing of meiosis progression from NEB to anaphase onset, both in *asp* and in colcemid-treated wild-type spermatocytes, seems to be largely unaffected, suggesting that the feeble spindle checkpoint of these cells (Rebollo and Gonzalez 2000; Savoian et al. 2000) is not triggered by the membrane-bound centrosomes' condition. The timing of the onset of noncentrosomal microtubule growth within the nuclear region in *asp* and colcemid-treated cells is tightly controlled. It occurs between 9 and 13 min after NEB, at the same time or marginally later than the end of chromosome condensation. If this process occurs with the same timing in wild-type cells, the noncentrosomal microtubules will intermingle with numerous centrosomal microtubules that are already present at this stage.

### Acentrosomal Microtubules Are Nucleated on the Inner Side of the Remnants of the NE and Not around the Chromosomes

**Figure 4:**
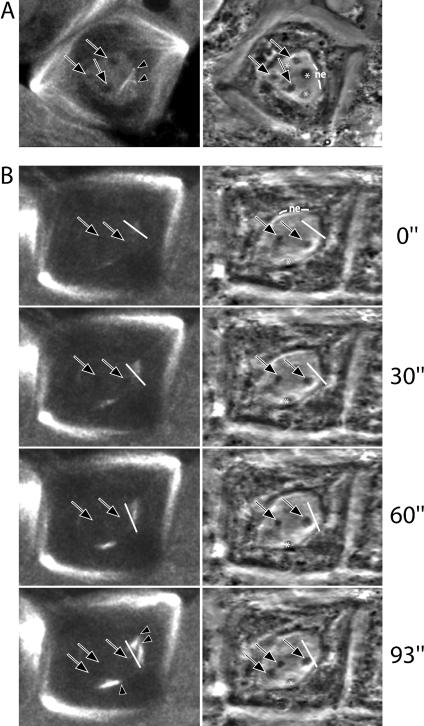
The Place of Noncentrosomal Microtubule Nucleation The initial stages of noncentrosomal microtubule nucleation revealed by an endogenous GFP–α-tubulin fusion (left) and phase contrast (right). Following the corresponding videos, it is possible to unmistakably tell the chromosomes (arrows) apart form the other phase-dark objects that are present over the nuclear region (asterisks). The cell in (A) is shown as a single timeframe and the cell in (B) as a time-lapse series. In both cells, noncentrosomal microtubule nucleation (arrowheads) takes place close to the remains on the NE and does not overlap with the major chromosomes. Nucleation sites can be clustered (A) or dispersed (B). In the time-lapse series (B), only the chromosomes that are in focus are labelled. Timepoint 0 min in these series corresponds to the first sign of noncentrosomal microtubule nucleation, around 11 min after NEB. A white bar marks the growing end of a microtubule bundle that at timepoint 93 min reaches one of the bivalents.

To determine the nucleation site of the microtubules organised over the nuclear region, we followed the initial stages of microtubule assembly by time-lapse microscopy, acquiring several Z series of XY confocal and phase-contrast sections at different timepoints. From these, we generated a time-lapse series of 3D reconstructions that allowed us to localise the foci of nucleation of anastral microtubules. We were able to draw the following three main conclusions that apply to both *asp* and colcemid-treated spermatocytes. Firstly, the foci from which microtubules grow may be clustered (Figure 4A) or dispersed (Figure 4B). Secondly, no significant correlation can be established between the site of microtubule nucleation and the chromosomes (Figure 4A and 4B). Finally, nucleation takes place in close proximity to the remnants of the NE (ten out of ten cells reconstructed; Figure 4A and 4B), which in *Drosophila* ruptures without disassembling completely (Tates 1971; Stafstrom and Staehelin 1984).

### The Anastral Spindles Organised in Cells with Membrane-Bound Centrosomes Can Sustain Some Degree of Chromosome Segregation

**Figure 5:**
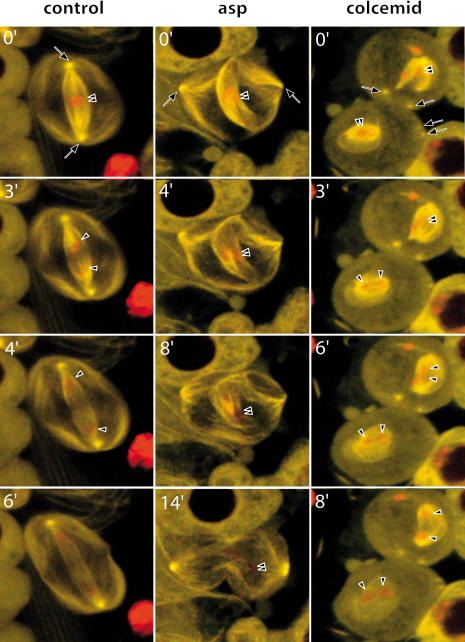
Chromosome Segregation in Anastral Spindles in *Drosophila* Spermatocytes (Control [Video 5]) At metaphase I (0), the bivalents (revealed by a His2Avd–YFP fusion, shown by double arrowheads) are aligned in the middle of the spindle (revealed by a GFP–α-tubulin fusion), at the metaphase plate. At the onset of anaphase (3 min), the homologue chromosomes start to migrate towards opposite poles (single arrowheads) and to decondense. During anaphase B (4 min and 6 min), the spindle poles move apart from each other and the two sets of decondensed chromosomes become further separated. (*asp* [Video 6]) At timepoint 0, the bivalents align at the metaphase plate. Homologue chromosomes split apart at the onset of anaphase I (4 min). However, anaphase A migration is highly impaired. By the time the chromosomes start to decondense, they have barely moved towards the spindle poles (8 min and 14 min), and often homologue chromosomes end up included in the same daughter nucleus. (Colcemid [Video 8]) As in *asp* spermatocytes, the asters (arrows) remain at the plasma membrane at metaphase I in colcemid-treated cells, and the bivalents align in a metaphase plate-like within the acentrosomal spindles (0 min). Homologue chromosomes split apart at the onset of anaphase (upper cell, 6 min) and significantly segregate from one another (upper cell, 8 min; lower cell, 3 min). Further separation of the daughter nuclei during anaphase B is very limited in these cells (8 min), and cytokinesis does not occur.

We then decided to study in more detail the extent to which the anastral spindles organised in cells with membrane-bound centrosomes can mediate successful cell division. To this end, we produced transgenic flies carrying a GFP–α-tubulin fusion together with a His2AvD–YFP (yellow fluorescent protein) strain so that both chromosomes and microtubules could be visualised in the same cell (Figure 5; Video 5). In *asp* cells, during prometaphase, the bivalents do not move to the extent that they do in control cells (data not shown). As mentioned before, congression occurs (Figure 5; Video 6), but orientation is rarely bipolar. Homologue chromosomes separate at the onset of anaphase, but they barely move, remaining near the center of the spindle. Moreover, they tend to cosegregate (Video 7) and end up included in the same daughter nucleus. All together, these abnormalities result in high levels of aneuploidy in agreement with previous genetic analysis data (Ripoll et al. 1985). In contrast, in half (52%) of the anastral spindles assembled following transient colcemid treatment, homologue chromosomes could be seen to segregate from one another (Figure 5; Video 8). Anaphase in these cells is not complete, however, because only the chromosome-to-pole movement (anaphase A) is observed. The further separation achieved in wild-type cells by the extension of the spindle (anaphase B) is very limited in these cells.

### The Orientation of the Cytokinesis Furrow Correlates with the Position of the Membrane-Bound Asters, Independently of Spindle Orientation

**Figure 6:**
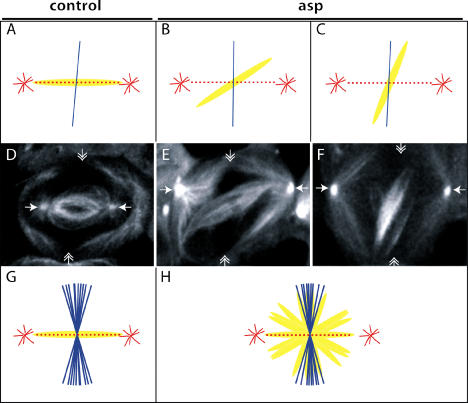
Correlation between the Orientation of the Cytokinesis Furrow, the Asters, and the Spindle in *asp* Spermatocytes Schematic representation (A–C) of the relative position of the asters (red), the spindle (yellow), and the cytokinesis furrow (blue), corresponding to a control cell (D) and two examples of *asp* mutant spermatocytes (E and F), respectively. Asters (arrows) and spindles are labelled with a GFP–α-tubulin fusion. The position of the cleavage furrow (double-headed arrow) was determined by time-lapse imaging of these cells (data not shown). In wild-type cells (*n* = 10), plotting spindle and furrow orientation relative to the interastral axes shows that asters and spindle are tightly aligned, and cleavage occurs at an angle of 90° ± 10° with respect to them (G). In *asp* spermatocytes (*n* = 10), the plane of cleavage occurs at a 90° ± 10° angle with respect to the asters and does not correlate with the orientation of the anastral spindle.

Following colcemid treatment, we have never observed complete cytokinesis. However, as reported before (Riparbelli et al. 2002), cytokinesis does proceed to completion in around half (47%, *n* = 19) of *asp* cells. These cells, which contain unconnected centrosomal asters and anastral spindles, provide a valuable experimental system to assess the contribution of asters and spindle to specifying the place of cleavage. To this end, we plotted the angles between the line defined by the two asters, the major spindle axis, and the plane of cleavage in wild-type and *asp* spermatocytes (Figure 6). Two conclusions can be drawn from these data. Firstly, the anastral spindles assembled in *asp* cells can be observed at any angle, even up to 90°, with respect to the position of the two asters. Interestingly, in most such cases, furrow progression forces the spindle to rotate and align with the asters so that, at the end, a fairly normal cytokinesis takes place. Secondly, the orientation of the plane of cleavage keeps a tight 90° ± 10° with respect to the axis defined by the asters and does not correlate with the orientation of the anastral spindle.

## Discussion

**Figure 7:**
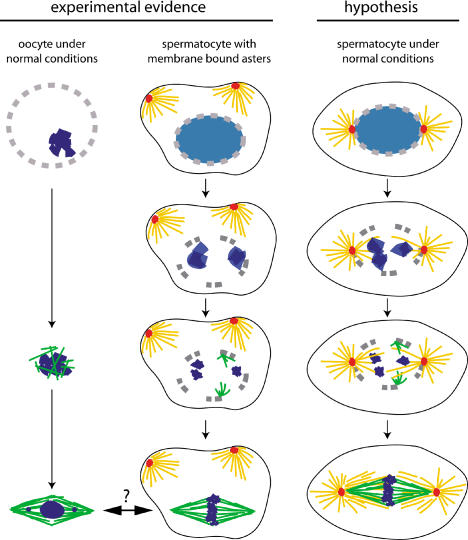
Noncentrosomal Microtubules and Spindle Assembly (Central column) Spindle assembly in *Drosophila* spermatocytes with membrane-bound centrosomes. At the time of NEB, the chromatin (pale blue) starts to condense, and the membrane-bound centrosomes (red) organise asters (yellow) at a significant distance from the nuclear region. Around 12 min after NEB, the first noncentrosomal microtubules (green) start to nucleate near the remnants of the NE (grey), as the chromosomes achieve full condensation (dark blue). These microtubules then bundle, associate with the chromosomes, and eventually end up organised into a bipolar anastral array whose shape is reminiscent of the female meiotic spindle. (Left column) Spindle assembly in wild-type *Drosophila* oocytes (Theurkauf and Hawley 1992; Matthies et al. 1996). NEB starts at the beginning of stage 13 of oocyte development. At this stage, the oocyte does not contain centrosomes and the chromosomes (karyosome) are tightly condensed (dark blue). Microtubules (green) appear 11–15 min after NEB within the nuclear region in association with the karyosome. These microtubules form bundles and are sorted around the chromatin into a bipolar spindle. Evidence suggests that ER components may be required for spindle assembly in these cells (Kramer and Hawley 2003). At metaphase I, recombined bivalents are aligned at the spindle equator, while those that have not recombined are found closer to the spindle poles. Meiosis remains arrested at this point (stage 14) until oocyte activation. Despite the obvious morphological similitude, the equivalence between these and the anastral spindles organised in spermatocytes with membrane-bound centrosomes is unclear. (Right column) Hypothesis regarding the contribution of centrosomal and noncentrosomal microtubules to spindle assembly during meiosis I in wild-type *Drosophila* spermatocytes. Before NEB, the centrosomes are located at opposite positions near the nucleus. Shortly after NEB, astral microtubules enter the nuclear region and make the first contact with the condensing chromatin. No evidence of noncentrosomal microtubule polymerisation near the nuclear region at this stage has been found yet. Once chromosomes are fully condensed, microtubule bundles of centrosomal origin (yellow) connecting centrosomes to chromosomes already exist. At this stage, noncentrosomal microtubules (green) start to polymerise in association with the remnants of the NE. These microtubules form bundles that interact with the chromosomes and intermingle with the microtubules of centrosomal origin. The fully mature spindle in these cells would therefore contain a spindle-shaped structure made of microtubules of noncentrosomal origin (green) embedded in another spindle-shape array made of two overlapping asters (yellow). We propose that each of these subsets may perform to a certain extent some of the functions carried out by normal spindles, but neither of them can on its own mediate robust cell division.

We have found that when the asters are kept near the plasma membrane during meiosis I in *Drosophila* spermatocytes, noncentrosomal microtubules appear over the nuclear region and, in a fraction of the cells, are sorted into anastral bipolar spindles (summarised in Figure 7). Identical observations are derived whether centrosomes are forced to remain membrane-bound by mutation in *asp* or by transient colcemid treatment of wild-type cells. The very different nature of these two experimental conditions strongly argues against these spindles being assembled as a consequence of the experimental conditions themselves. It rather suggests that the observation of anastral spindles is due to the impaired ability of the plasma membrane-bound centrosomes to contribute to spindle assembly. Anastral spindles are also assembled following the removal of centrosomes by laser ablation or microdissection in cultured cells (Khodjakov et al. 2000; Hinchcliffe et al. 2001) or by inhibiting the formation of centrosomes in mutant *Drosophila* embryos (Megraw et al. 1999; Vaizel-Ohayon and Schejter 1999), thus reinforcing this argument.

### The Place of Noncentrosomal Microtubule Nucleation

Noncentrosomal microtubule nucleation during cell division is thought to take place over the chromatin (Nachury et al. 2001). This assumption is largely based on the observations carried out in the few acentrosomal systems in which spindle assembly has been followed by time-lapse microscopy (reviewed in Karsenti and Vernos 2001). These include wild-type *Drosophila* female meiocytes (Theurkauf and Hawley 1992; Matthies et al. 1996), parthenogenetic *Sciara* embryos (de Saint Phalle and Sullivan 1998), and *Xenopus* egg extracts (Heald et al. 1996). It is also consistent with the active role of chromosomes in spindle organisation. For instance, it has been reported that bivalents micromanipulated away from the spindle in *Drosophila* spermatocytes induce the assembly of anastral minispindles in the cytoplasm (Church et al. 1986). Likewise, the removal of chromosomes before NEB has been shown to inhibit spindle assembly in grasshopper spermatocytes (Zhang and Nicklas 1995), although the phenotype of *fusolo* mutants, recently described, seems to argue otherwise (Bucciarelli et al. 2003). Moreover, the chromosomal localisation of the RanGEF RCC1 is expected to result in a local enrichment of the GTP-bound form of Ran, known to facilitate spindle assembly (Nachury et al. 2001; Wilde et al. 2001; Gruss et al. 2002; reviewed in Hetzer et al. 2002), thus providing a mechanistic interpretation for the suspected role of chromatin in this process.

In contrast, our observations reveal that nucleation of the noncentrosomal microtubules over the nuclear region occurs over the remnants of the NE, which in *Drosophila* are present throughout cell division despite extensive fenestration at the onset of prometaphase (Tates 1971; Stafstrom and Staehelin 1984; Church and Lin 1985). However, upon closer examination, our observations may also be consistent with the literature quoted above. The single bivalents that organise minispindles when micromanipulated into the cytoplasm in *Drosophila* spermatocytes have actually been shown to be surrounded by masses of stacked membranes, whose contribution to microtubule nucleation/stabilisation, according to the authors themselves, cannot be ruled out (Church et al. 1986). In *Drosophila* oocytes, too, the meiotic spindle is ensheathed in a membrane structure derived from the endoplasmic reticulum (ER). Although the bulk of microtubules has been described by time-lapse confocal microscopy to form over the chromatin (Theurkauf and Hawley 1992; Matthies et al. 1996), the contribution of these membranes to the initial stages of microtubule nucleation cannot be discarded either. In this regard, the recent cloning of *Axs*, which encodes a transmembrane protein associated with the membranes that surround the spindle and is required for the segregation of achiasmate chromosomes, is very tantalising (Kramer and Hawley 2003). Asx is distributed within the ER of the germinal vesicle just before meiotic spindle assembly. Upon germinal vesicle breakdown (GVBD), Axs associates with the developing spindle through all stages of assembly. These observations have been taken as an indication that the ER may be organised into structures that impinge on spindle assembly during meiosis in *Drosophila* females (Kramer and Hawley 2003), very much in line with our observations in *Drosophila* spermatocytes.

Indeed, our results do not discard the contribution of chromatin to microtubule stabilisation and sorting into a bipolar array, even if chromatin itself is not the place of initial microtubule nucleation, nor do they rule out the possibility of microtubules being polymerised over the chromatin at later stages. They merely show that in these cells, the remaining bits of the fenestrated NE provide a particularly favourable environment to sustain the initial stages of noncentrosomal microtubule nucleation. Moreover, these observations strongly suggest that, unlike centrosomes, the foci of microtubule nucleation over the nuclear region do not behave as stable MTOCs. As soon as the microtubule bundles acquire a certain length, they interact with the condensed chromosomes and are often sorted into a bipolar spindle, regardless of the initial number of nucleation sites. We have not been able to detect γ-Tub23C at these nucleation sites.

We still do not know the actual contribution of Ran to spindle assembly in *Drosophila* spermatocytes, although, given its known conservation across distant species (Hetzer et al. 2002), it is likely to play a major role. Since orthologues of most of the known components of this pathway are known in *Drosophila*, it is technically possible to address this question both under normal conditions and in cells in which centrosomes cannot contribute to spindle assembly as described in this work. Experiments are underway in our laboratory to address these points.

### Functional Relevance of the Anastral Spindles

Perhaps the most fundamental question regarding the anastral spindles organised in cells that normally contain centrosomes is the extent to which they could provide a back-up, able to mediate successful and robust cell division when the centrosomes cannot contribute to spindle assembly. Our observations suggest that this is an unlikely scenario. Firstly, only a fraction of cells display a single bipolar array, the rest being accounted for by cases in which either the spindle is multipolar or there is more that one per cell or there is no spindle at all. Secondly, chromosome segregation is also significantly less efficient than in control cells. These two points, however, carry a caveat since they could reflect the effect of depleted *asp* function or residual traces of active colcemid, rather than the anastral nature of the spindle. Finally, cytokinesis is severely disrupted in these cells. Around half of *asp* spermatocytes containing anastral spindles go through and complete cytokinesis. However, in these cells, the orientation of the cleavage furrow correlates tightly with the position of the two asters and not at all with the orientation of the spindle. This situation gives rise to cases in which the plane of cleavage is nearly parallel to the spindle. In colcemid-treated cells that contain notoriously small asters, cytokinesis does not occur. These observations strongly argue that asters contribute to specify the place of furrow and may be required for cleavage. The contribution of centrosomes to ensure proper cytokinesis has been previously observed in vertebrate cell lines (Rieder et al. 1997; Savoian et al. 1999; Hinchcliffe et al. 2001; Khodjakov and Rieder 2001), human cell lines (Gromley et al. 2003), *Dyctiostelium* (Neujahr et al. 1998), or *Xenopus* (Takayama et al. 2002). This conclusion, however, is not consistent with the observation that cytokinesis is not inhibited in asterless *Drosophila* spermatocytes (Bonaccorsi et al. 1998).

Therefore, the anastral spindles organised in spermatocytes with membrane-bound centrosomes seem able to provide only some of the functions required for cell division, with relatively low efficiency. The functionality of the anastral spindles assembled in embryos laid by *cnn* mutant females, which do not appear to contain centrosomes, is also compromised. These spindles are not always properly shaped, the chromosomes are not tightly aligned at the spindle equator, chromosome movements are nonsynchronous, and their segregation not always faithful (Megraw et al. 1999; Vaizel-Ohayon and Schejter 1999). Thus, in this instance, too, when centrosome function is abrogated in a syncytium that normally contains centrosomes and that does not naturally undergo parthenogenesis, anastral spindles can be assembled that are able to perform some of the functions of their wild-type counterparts, but in a rather inefficient manner.

### Origin of the Anastral Spindles: Neomorphic or Constitutive

Two alternative interpretations can account for the origin of the anastral spindles that we have observed (Khodjakov et al. 2000). First, they could be neomorphic structures, assembled through a pathway normally repressed that is only triggered in response to the impaired contribution of centrosomal microtubules. Although we cannot at the moment discard this interpretation, we find it hard to envisage how such an alternative pathway could have evolved, given the extremely low frequency of centrosome loss or inactivation in wild-type populations. Moreover, it is also difficult to imagine what sort of signalling mechanism could trigger the alternative pathway in these cells since centrosomes are still present and active as MTOCs.

Alternatively, these anastral microtubule arrays could be a constitutive component of wild-type spindles, normally masked by the abundance of centrosome-derived microtubules, but revealed when asters are kept away. This interpretation is summarised in Figure 7. In wild-type spermatocytes under normal conditions, the first astral microtubules enter the nuclear area shortly after NEB and start to build a bipolar spindle as chromosome condensation progresses. By the time chromosome condensation is fully achieved, a distinct bipolar spindle can be observed in these cells. However, it is not yet fully mature, as the number of microtubules will still increase until anaphase onset. It is about this time that nucleation of the acentrosomal microtubules occurs in cells with plasma membrane-bound centrosomes. Therefore, if this process occurs at the same time in wild-type cells, the acentrosomal microtubules could significantly contribute to the maturation of the cell division spindle. This interpretation is consistent with the recent proposal put forward by Gruss et al. (2002) to account for their observations regarding spindle assembly in HeLa cells. They found that when the function of the human homologue of TPX2 is inhibited by RNA interference, the centrosomal asters do not interact and do not form a spindle. From these observations, they concluded that, intermingled with microtubules of centrosomal origin, the mitotic spindle may contain noncentrosomal microtubules that are stabilised and organised by the chromatin and are essential for the assembly of functional spindles. In *Drosophila*, secondary spermatocytes' mutation in *fusolo* seem to reveal the centrosome-derived component of the spindle (Bucciarelli et al. 2003). Forcing the asters away from the nucleus in *Drosophila* primary spermatocytes reveals the noncentrosomal component that, indeed, does not require asters to get organised into a spindle-like structure. We propose that both components are required to mediate robust cell division. The very recent finding of peripheral, noncentrosomal microtubules that contribute to spindle assembly in LLCPK1α cells provides additional evidence to substantiate this conclusion (Tulu et al. 2003).

Regardless of their neomorphic or constitutive nature, the acentrosomal spindles that we have found in *asp* and colcemid-treated spermatocytes are, from a morphological point of view, closely reminiscent of the anastral female meiotic spindles found in many animal species, including *Drosophila* (Theurkauf and Hawley 1992). The same holds true for the anastral spindles assembled when centrosomes are removed from the cell or cannot be organised due to mutation in essential centrosomal components (Megraw et al. 1999; Khodjakov et al. 2000; Khodjakov and Rieder 2001; Hinchcliffe et al. 2001). In the case of the *Drosophila* female meiotic spindle, the timing of microtubule nucleation is also very similar: between 9 and 12 min after NEB in spermatocytes and 11 to 15 min in oocytes (Matthies et al. 1996). These similarities have led some to propose that experimentally induced anastral spindles could require the same motors and structural components that build the spindles in female meiocytes (Megraw et al. 1999; Khodjakov et al. 2000) (summarised in Figure 6). In fact, it has been suggested that the absence of some of these components at the time syncytial divisions occur could explain the lack of robustness of the anastral spindles assembled in embryos derived from *cnn* mothers (Megraw et al. 1999). We still do not know to what extent the anastral spindles of spermatocytes share components with the oocyte spindle. Some essential ones cannot be shared, though, since they are only expressed in the female germline. Given the wealth of probes and mutants available in *Drosophila*, it should be possible to draw a clear picture of the situation regarding this fundamental question.

## Materials and Methods

### 

#### Fly stocks

Flies from *w^1118^;e^11^ asp^E3^/TM6C* and *w^1118^;red asp^L1^/TM6C* stocks were crossed to generate *w^1118^;e^11^ asp^E3^/red asp^L1^* transheterozygous individuals. The viability of *asp^E3^/asp^L1^* males is high, but they are poorly fertile and produce high levels of aneuploid gametes.

#### Transgenes

The chromosomes were labeled with transgenes expressing either a His2avD–GFP fusion (Clarkson and Saint 1999) or its derivative, His2avD–EYFP, constructed by us under the control of the polyubiquitin promoter (Lee et al. 1988). To visualise centrioles, we used the transgene expressing GFP-PACT (pericentrin-AKAP450 centrosomal targeting) (kindly provided by J. Raff) that contains the predicted Drosophila homologue of the PACT domain described by Gillingham and Munro (2000). To visualise microtubules, we constructed a transgene that contained the GFP–α-tub84B fusion as previously described (Grieder et al. 2000) under the control of the polyubiquitin promoter (Lee et al. 1988).

#### Time-lapse recording

Live spermatocytes were recorded as previously described (Rebollo and Gonzalez 2000, 2003). For most applications, we collected a series of timepoints at 15–30 s intervals, each containing four to eight XY sections at different depths along the Z axis and including both the phase-contrast and fluorescence channels. For more-detailed 3D reconstruction, stacks containing 20 sections were obtained. Laser intensity was always kept to a minimum, and only the excitation laser line 488 was utilised. GFP and YFP signals were distinguished by overlying the two recorded channels. Image processing was performed with NIH-Scion Image, Interactive Data Language (IDL), and huygens2. For 3D reconstructions, we wrote macros in NIH-Scion Image to navigate through the three dimensions of the cell stack.

#### Colcemid treatment

Newly hatched adult males were fed for 8–12 h with a solution containing 32 μg/ml of colcemid (Sigma, St. Louis, Missouri, United States) in 1 M sucrose. Upon dissection, their testes were prepared for in vivo imaging as described above. Once under the microscope, microtubules were allowed to repolymerise by a 1-s pulse of 350 nm light that inactivates the drug. For simplicity, this entire procedure of exposure to colcemid followed by light inactivation of the drug is referred to through this manuscript as ‘colcemid treatment.’

## Supporting Information

### Accession Numbers

The FlyBase accession numbers discussed in this paper are α-tubulin 84B (CG1913), *asp* (CG6875), *Axs* (CG9703), *cnn* (CG4832), γ-Tub23C (CG3157), His2AvD (CG5499). The GeneBank accession numbers discussed in this paper areGFP (U57609.1), Ran (NM_006325), RCC1 (D00679), TPX2 (BC020207), and YFP (U57609.1).

## 

**Video 1:** Centriole Migration in Primary Spermatocytes (849 KB MOV)

**Video 2:** Spindle Assembly in Control Spermatocytes (442 KB MOV)

**Video 3:** Spindle Assembly in *asp* Spermatocytes (675 KB MOV)

**Video 4:** Spindle Assembly in Colcemid-Treated Spermatocytes (844 KB MOV)

**Video 5:** Chromosome Segregation in Control Spermatocytes (206 KB MOV)

**Video 6:** Chromosome Segregation in *asp* Spermatocytes I (452 KB MOV)

**Video 7:** Chromosome Segregation in *asp* Spermatocytes II (377 KB MOV)

**Video 8:** Chromosome Segregation in Colcemid-Treated Spermatocytes (465 KB MOV)

## Abbreviations

ER: endoplasmic reticulum
GFP: green fluorescent protein
GVBD: germinal vesicle breakdown
IDL: Interactive Data Language
MTOC: microtubule organising centre
NE: nuclear envelope
NEB: nuclear envelope breakdown
PACT: pericentrin-AKAP450 centrosomal targeting
PCM: pericentriolar material
YFP: yellow fluorescent protein
